# A visible-light-controlled platform for prolonged drug release based on Ag-doped TiO_2_ nanotubes with a hydrophobic layer

**DOI:** 10.3762/bjnano.9.170

**Published:** 2018-06-14

**Authors:** Caihong Liang, Jiang Wen, Xiaoming Liao

**Affiliations:** 1 College of Materials Science and Engineering, Sichuan University, Chengdu, Sichuan 610065, China

**Keywords:** Ag doping, drug delivery, hydrophobic layer, prolonged drug release, TiO_2_ nanotubes, visible-light-controlled release

## Abstract

In this work, a visible-light-controlled drug release platform was constructed for localized and prolonged drug release based on two-layer titania nanotubes (TNTs) fabricated using by an in situ voltage up-anodization process. The visible-light photocatalytic activity is improved by loading Ag onto the TNTs by NaBH_4_ reduction. Then, the TNTs containing Ag nanoparticles were modified with dodecanethiol (NDM) to create a hydrophobic layer. To demonstrate the visible-light-controlled drug release, the Zn^2+^ release behavior of the samples was investigated. In the initial 12 h, TNTs without NDM displayed a faster release rate with 29.4% Zn^2+^ release, which was more than three times that of the TNTs with NDM (8.7% Zn^2+^ release). Upon visible-light illumination, drug release from the sample coated with NDM was shown to increase due to the photocatalytic decomposition of NDM. The amount of released Zn^2+^ for this sample increased up to 71.9% within 12 h, indicating visible-light-controlled drug release. This drug release system may exhibit promising application as a localized, prolonged drug delivery platform.

## Introduction

Titanium dioxide nanotubes (TNTs) are often employed as drug carriers, owing to their well-defined geometry, stable structure [1], high surface-to-volume ratio, highly hydrophilic wetting characteristic [2–3], and remarkable photocatalysis properties [4]. However, the traditional drug delivery systems still exhibit many obvious drawbacks such as low drug solubility, uncontrolled distribution and systemic toxicity towards non-target tissues [5]. As a result, in order to improve their drug-release ability for prolonged or controlled drug delivery, numerous methods including chemical and structural modification have been applied to modify the surface of TNTs [6–9].

By altering the anodizing voltage and time, some distinctive nanotube structures have been obtained, including multilayer [10], bamboo-type [11], branched tubes [12], and double-walled tubes [13]. Multilayered TNTs are known as the most controllable and useful drug delivery system [14]. In a previous work, Shi et al. [15] created two-layer TNTs with a pear-like structure which has a smaller upper layer diameter (60–80 nm) and a larger lower layer diameter (180–200 nm) by a novel in situ voltage up-anodization method to prolong the release of drugs. Gulati et al. [16] used an altered periodic voltage in the anodization process to produce the periodically shaped TNTs. The structural modification extended the effectiveness of the drugs for a longer period of time and avoided the burst release phenomena known to occur in the initial stage. Nevertheless, many of the side effects, such as systemic toxicity, still need to be solved and the effects of prolonging and slowing the release still need to be improved. Also, the drug dosage should be able to be directed to a specific location at a specific time. In particular, some points of high practical application related to drug delivery mechanisms are external factors, such as pH [17–19], illumination [14,19], ionic strength [20–21], and temperature [22–24]. Song et al. [14] fabricated an amphiphilic TNT structure to control the release of drugs. By using the hydrophobic organic portion to decorate the upper layer, nanotubes could hinder the hydrophilic liquid from getting into tubes and removing the drugs from the tubes and linkers. Making use of its excellent photocatalytic properties, the organic layer could then be destroyed, allowing the drugs to escape from the linkers and tubes more easily and quickly. Nevertheless, only 2–4% of solar energy is comprised of UV light, which can cause the denaturation or decomposition of some biomolecules and organic matter. As a result, such a trigger mechanism may not be the ideal way in an environment with adequate solar or natural light. Xu et al. [25] found a solution for enhancing the utilization of natural light by doping AuNPs onto the nanotubes to improve the photocatalysis of the TNTs, which feasibly allows drug release under visible light. However, controlled drug release in combination with extended release delivery via a TiO_2_ nanotube platform has been rarely investigated.

As a trace element essential to human being, previously published work [26] showed that Zn can greatly improve cell functionality by enhancing osteoblast proliferation and mineralization and promoting mesenchymal stem cell osteogenic differentiation. Meanwhile, its anti-inflammatory properties have also drawn wide attention. Thus, Zn exhibits significant, promising application in the biomedical field. Hence, Zn was selected as a model drug to be loaded into the TiO_2_ nanotubes for the drug delivery system in this work.

In present work, we provide an approach for a visible-light-controlled drug release platform based on the structural modification of TiO_2_ nanotubes. We decorated AgNPs onto two-layer TNTs fabricated by an in situ voltage up-anodization technique to improve the availability of visible light. In addition, we coated a hydrophobic organic layer (dodecanethiol) directly onto the hydrophilic surface of the nanotubes, which were preloaded with hydrophilic Zn^2+^-based drugs. Through this method, Zr-based drug release can be inhibited by the organic layer under the condition of darkness and can be promoted under the condition of visible light. Consequently, this release process can be considerably prolonged in the dark because of the existence of the hydrophobic layer and the smaller opening at the top of the TNT layer. Furthermore, the highly controlled release of drugs can be achieved by applying visible light, while the drug delivery process can be prolonged by the smaller opening of top layer.

## Results and Discussion

### Morphology, phase composition and wettability

**Figure 1:**
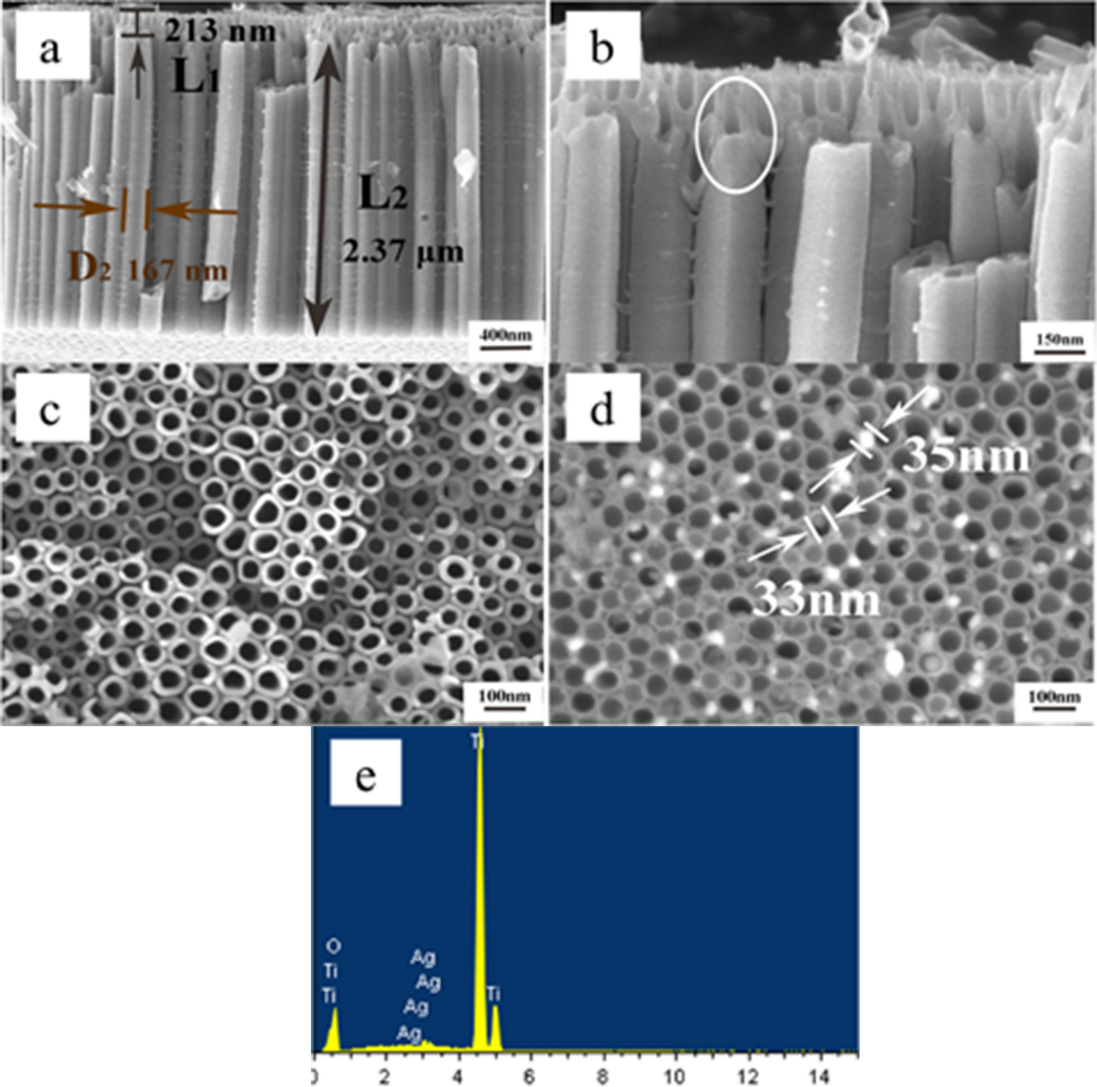
(a–d) SEM images of pear-shaped titania nanotubes. (a,b) Cross-sectional morphology (the white circle in b shows the connection between the first and second layers where they are integrated) and (c) surface morphology without Ag nanoparticles and (d) with Ag nanoparticles. (e) Energy dispersive X-ray spectrum of titania nanotubes with Ag nanoparticle (intensity vs energy (eV).

The cross-section and surface morphology of the two-layer nanotubes with and without AgNP loading and the corresponding energy dispersive X-ray spectrum (EDS) spectrum of the Ag-loaded sample are shown in Figure 1. Figure 1a presents the cross-section morphology of two-layer TiO_2_ nanotubes arrays, where the length of the second layer (2.37 μm) is longer than that of the first layer (213 nm) and the diameter of the second layer (167 nm) is larger than that of the first one (80 nm). The shape of the nanotubes in this work shows a similar pear-like structure with structures from our previous work under the same voltage processing conditions. Moreover, almost the same dimensions for the two-layer TiO_2_ nanotubes arrays were obtained, where the nanotube diameter of the first layer and the second layer was about 60–80 nm and 180–200 nm, respectively, and the length of the first layer was 400 nm (which is shorter than the length of the second layer at 2.06 μm) [15].

Elemental Ag was detected in the EDS spectrum of Ag-TNTs (Figure 1e) on the surface of the TNTs, suggesting that the Ag NPs were successful doped into the TiO_2_ nanotubes. From Figure 1d it can be seen that some particles, having diameters in the range of 30–40 nm, attach to the surface of the TNT arrays as well as the interior of the nanotubes.

**Figure 2:**
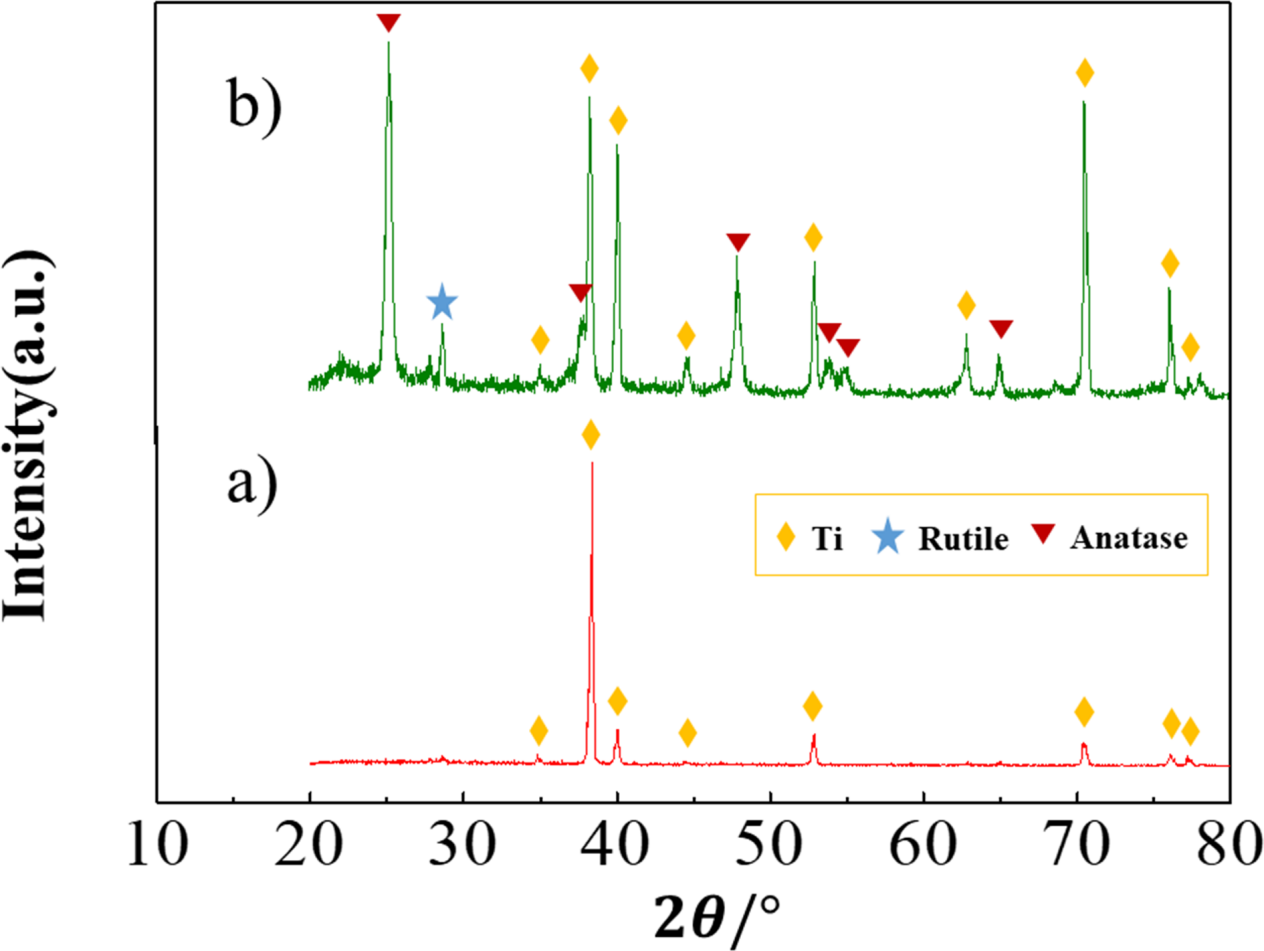
X-ray diffraction patterns of different samples: titania nanotubes before (a) and after (b) heat treatment, which is implemented after the anodization step.

X-ray diffraction (XRD) patterns (Figure 2) present the phase composition of the produced TNTs before (Figure 2a) and after (Figure 2b) heat treatment. Only the peaks of titanium can be observed for the sample before heat treatment in Figure 2a. On the contrary, it can be observed that the peaks of anatase phase appear for the samples shown in Figure 2b while the weak peak of rutile appears at around 28.7° (marked by blue stars).

**Figure 3:**
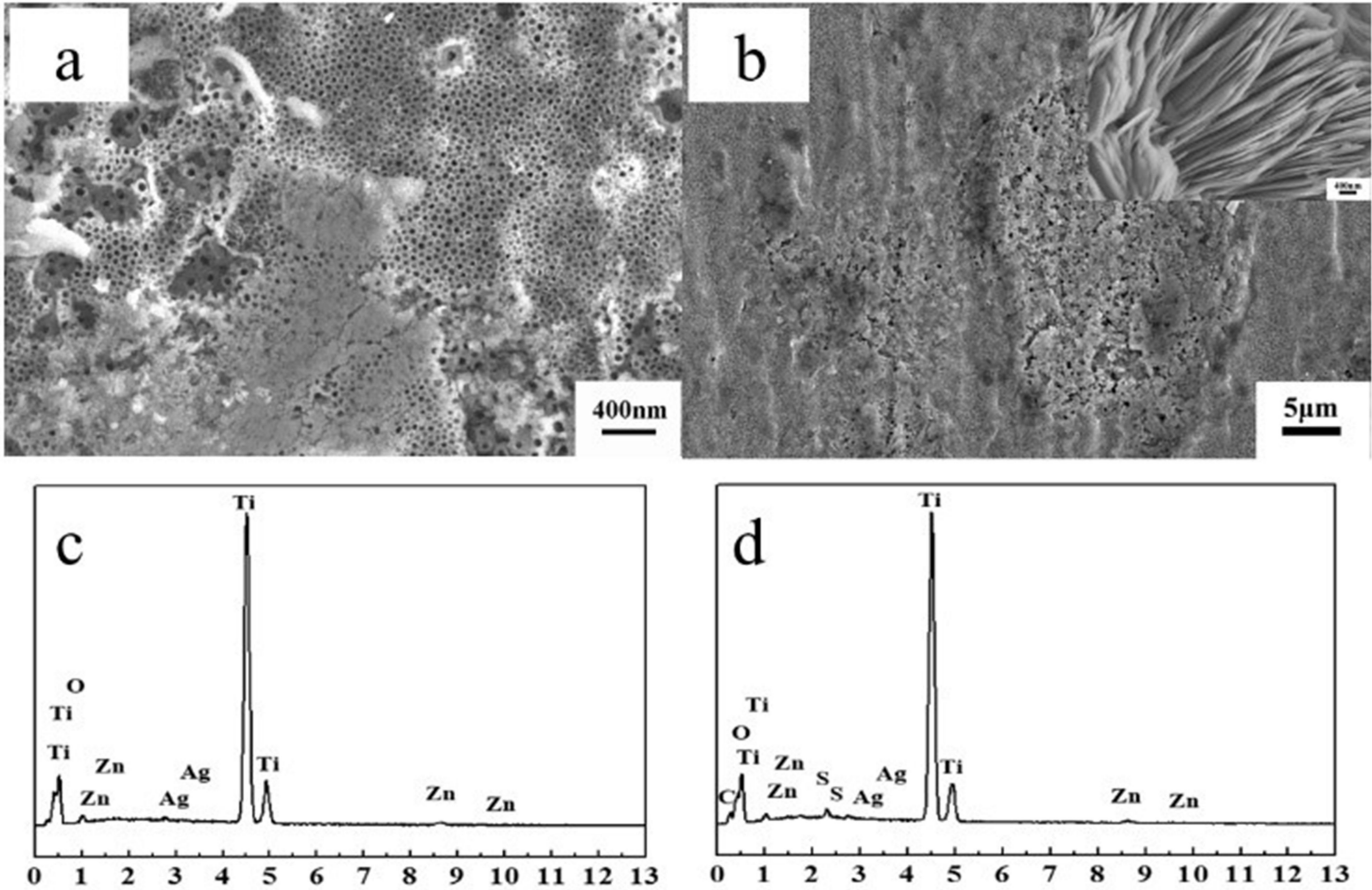
SEM images of different samples: (a) Zn-Ag-TNTs; (b) NDM-Zn-Ag-TNTs (the inset picture is part of the magnified zone of the NDM-Zn-Ag-TNT sample). The corresponding EDS spectra for samples: (c) Zn-Ag-TNTs and (d) NDM-Zn-Ag-TNTs (intensity vs energy (eV)).

**Figure 4:**
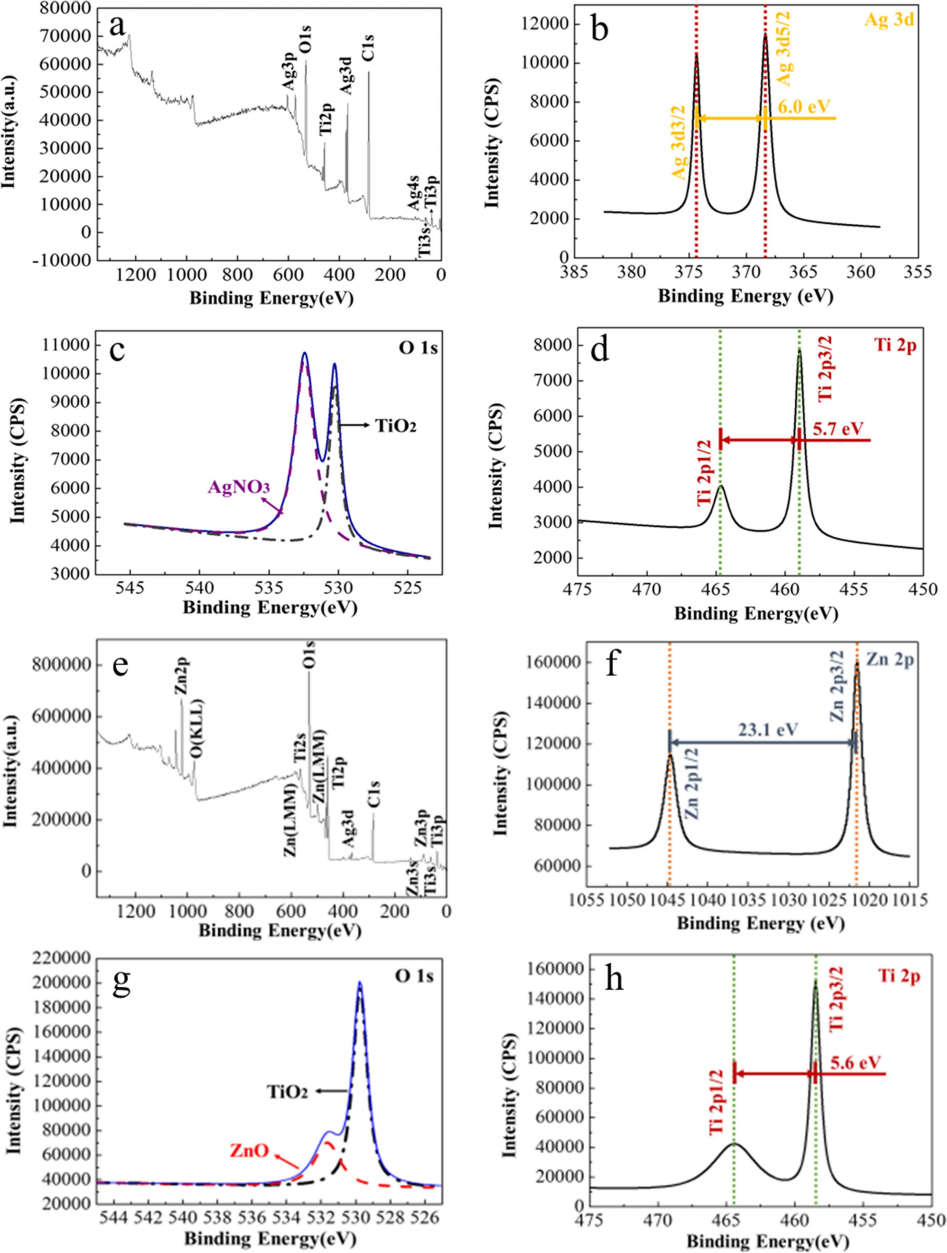
(a) The full XPS spectra, (b) the fine Ag 3d XPS spectra, (c) the fine O 1s XPS spectra and (d) the Ti 2p XPS spectra of Ag-TNTs. (e) The full XPS spectra, (f) the fine Zn 2p XPS spectra, (g) the fine O 1s XPS spectra and (h) the Ti 2p XPS spectra of Zn-Ag-TNTs.

The loading of Zn^2+^ and the NDM hydrophobic layer was verified by SEM and EDS; the results are shown in Figure 3. Furthermore, X-ray photoelectron spectroscopy (XPS) was employed to analyze the chemical state of the coating layer of Ag-TNTs and Zn-Ag-TNTs shown in Figure 4. Figure 3a,b clearly demonstrates that some particles and flakes are coated on the surface of nanotube arrays while Figure 3c and Figure 3d both illustrate that the covered matters mainly include Zn, Ti, Ag and O, suggesting that Zn has been loaded effectively onto the TNTs. Besides, elemental C and S are shown in EDS spectrum in Figure 3d as well. The existence of S demonstrates that NDM has been successfully applied onto the TNTs. The main components of the Ag and Zn substances were measured through XPS (see Figure 4). Figure 4a presents the XPS full spectrum of Ag-TNTs where Ag, Ti and O elements are shown to exist, while Figure 4e is the full XPS spectrum of Zn-Ag-TNTs, in which Zn, Ti, Ag and O elements are detected. These spectra suggest the successful decoration of Ag as well as loading of Zn. The presence of C may be attributed to the incorporation from the electrolyte and the organic contamination adsorbed from environment. Figure 4b shows the Ag 3d doublets with binding energies of 368.1 eV and 374.1 eV, corresponding to Ag 3d_5/2_ and Ag 3d_3/2_, respectively. According to the literature [27–28], these peaks can be attributed to metallic silver (Ag^0^). Figure 4d and Figure 4h show the high-resolution XPS spectra of Ti 2p of Ag-TNTs and Zn-Ag-TNTs. The peaks at the binding energy located at 458.6 eV in Figure 4d and Figure 4h are assigned to Ti 2p_3/2_, while that at 464.3 eV in Figure 4d and the one at 464.2 eV in Figure 4h belong to Ti 2p_1/2_, respectively. The splitting between Ti 2p_3/2_ and Ti 2p_1/2_ is 5.7 eV (Figure 4d) and 5.6 eV (Figure 4d), which correspond to the normal Ti^4+^ state. Additionally, the Zn peaks (Figure 4f) with the binding energy positioned at 1021.8 eV and 1044.9 eV were attributed to the Zn 2p_3/2_ and Zn 2p_1/2_ peaks, respectively. The separation between the Zn 2p doublet was 23.1 eV, illustrating that Zn mainly exists in form of ZnO, which is consistent with the results of Zhang [29] and Wang [30]. Figure 4c and Figure 4g show the high-resolution O 1s spectra. As it can be seen, the asymmetrical peak shape indicates the chemical state of O in samples is more than one state, which is deconvoluted by using symmetric Gaussian curves. The lower binding energy (529.6 eV) is ascribed to the oxygen atoms of TiO_2_ in Figure 4c and Figure 4g, while the additional peak at 532.2 eV is assigned to the residue reagent, AgNO_3_ [31] in Figure 4c and the peak in Figure 4g at 532.1 eV is usually attributed to the oxygen atoms of ZnO.

**Figure 5:**
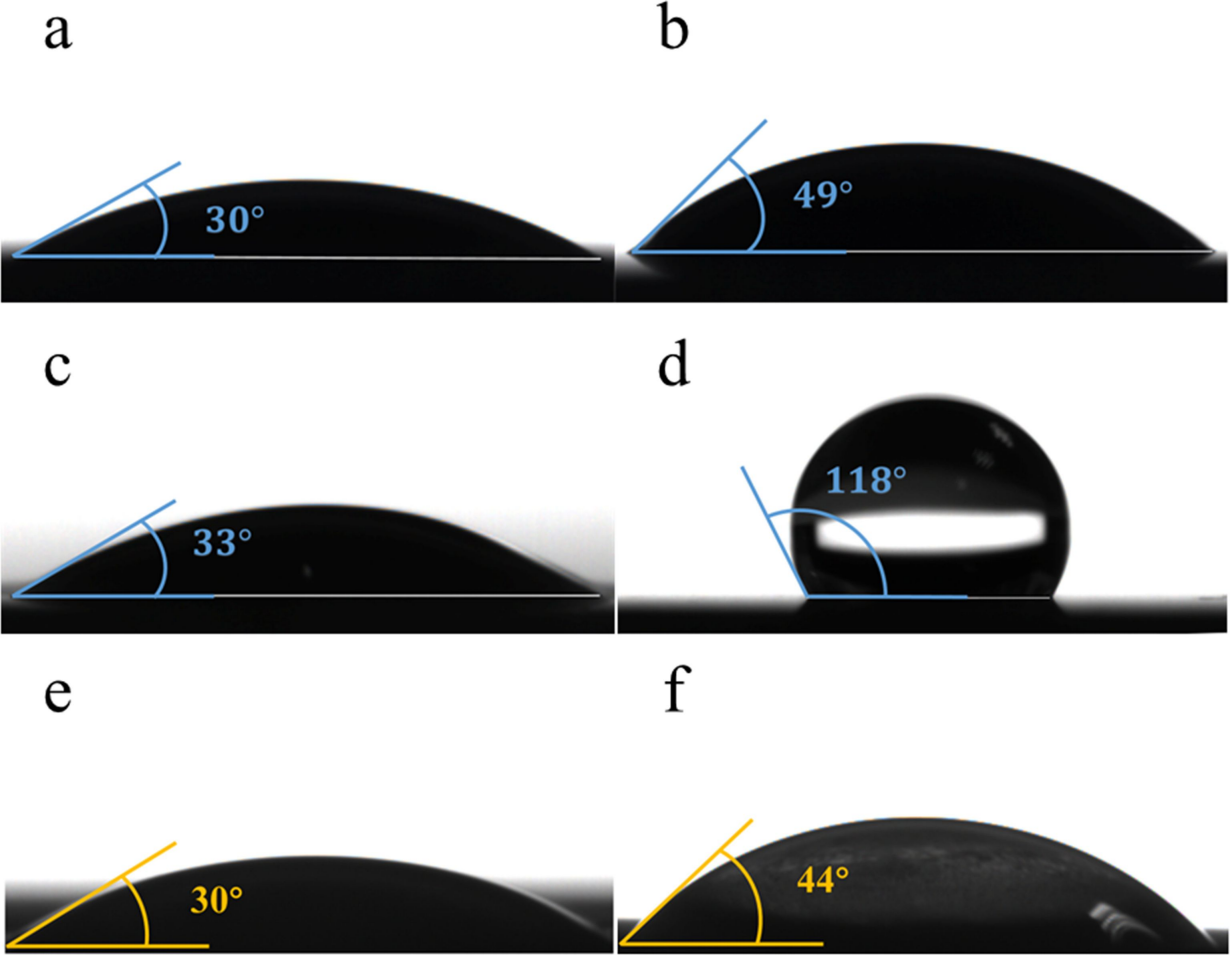
Optical images of a water droplet on TiO_2_ nanotube layers before drug release using: (a) bare TNTs, (b) after doping the TNTs with Ag nanoparticles, (c) after loading ZnO onto the structures, and (d) after coating the structures with NDM. Optical images of a water droplet on TiO_2_ nanotube layers after drug release with: (e) Zn-Ag-TNT sample upon irradiation and (f) NDM-Zn-Ag-TNT sample upon irradiation.

Figure 5 demonstrates the water contact angles (CAs) of the different treated samples. As-grown TiO_2_ nanotube arrays presents total hydrophilicity with a water CA of 30° (Figure 5a) without any surficial alteration [2–3]. Besides, TNTs modified with Ag (Figure 5b) and ZnO-Ag (Figure 5c) show hydrophilicity as well. Meanwhile, the CAs of modified TNTs increased slightly by 19° after decoration with Ag and then decreased by 16° due to the loading of the Zn^2+^-based drug. Furthermore, organic mutilayers with functional groups, including carboxylates or silanes [32], can be used to modify the surface of nanotubes. In the present work, NDM was chosen to alter the hydrophilic layer to hydrophobic by attaching it to the Zn-Ag-TNTs material. The contact angle of NDM-Zn-Ag-TNTs (118°) in Figure 5d demonstrates that the surface wettability of nanotubes does transform from hydrophilic to hydrophobic. Meanwhile, the wettability of the TNTs after a 17-day drug release test was also demonstrated (shown in Figure 5e,f). Figure 5e illustrates that the CA of TNTs without NDM is equal to that of bare TNTs (CA = 30°). The CA of sample NDM-Zn-Ag-TNTs was 44° as shown in Figure 5f under illumination in comparison with the primary CA of 118°, revealing the photocatalytic decomposition of the NDM layer. Also, the property of these nanotubes has transformed from hydrophobic to hydrophilic. The results show that NDM is successfully coated on the TNTs and the decomposition process of NDM can be enhanced by visible light.

### Characterization of light absorbance

**Figure 6:**
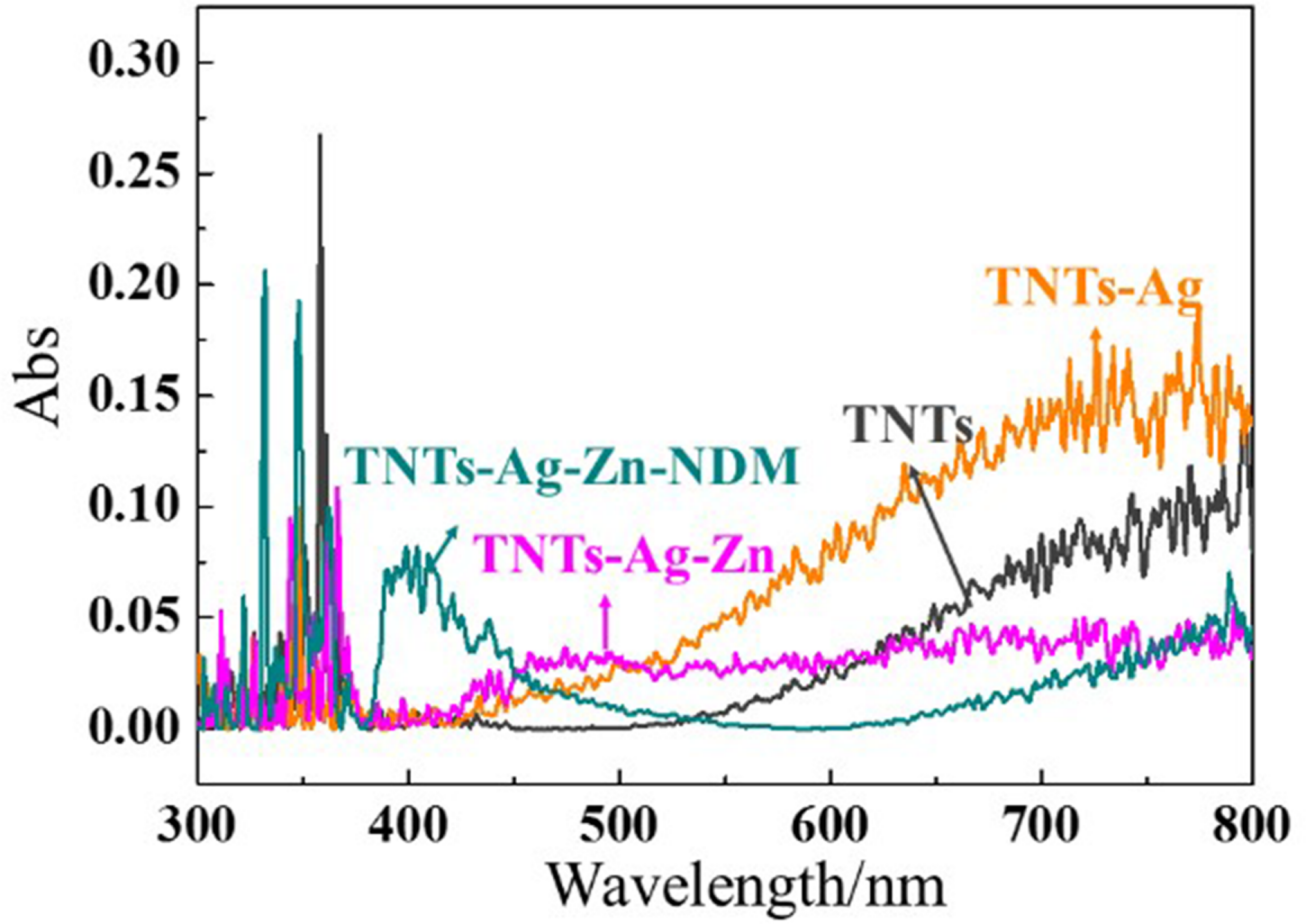
The absorbance spectra of TNTs (dark gray line), Ag-TNTs (orange line), Zn-Ag-TNTs (magenta line) and NDM-Zn-Ag-TNTs (dark cyan line).

The absorbance spectra of TNTs, Ag-TNTs, Ag-Zn-TNTs and Ag-Zn-NDM-TNTs samples in the range of 300–800 nm are shown in Figure 6. The dark gray, orange, magenta and dark cyan lines correspond to the light absorption of the TNTs, Ag-TNTs, Zn-Ag-TNTs and NDM-Zn-Ag-TNTs samples, respectively. Obviously, the absorbance of TNTs decorated with Ag is higher than bare TNTs within the visible light range (450–800 nm). The loading of AgNPs promotes surface plasmon resonance (SPR) scattering into the TNT layer, which further increases the nearby electric field [33] and results in the generation of e^−^ and h^+^ even under irradiation with lower energy light. Thus, doping with AgNPs has evidently enhanced the TiO_2_ photocatalysis under visible-light irradiation. However, after loading with the Zn^2+^-based drug and coating with the NDM layer, the absorbance of the Zn-Ag-TNTs and NDM-Zn-Ag-TNTs samples are almost lower than that of Ag-TNTs. For the Zn-Ag-TNTs sample, its absorbance is higher than bare TNTs at wavelengths below 619 nm, while its absorbance is lower than the TNTs at wavelengths greater than 619 nm. In comparison with bare TNTs, the absorbance of NDM-Zn-Ag-TNTs is higher in the range 450–533 nm, whereas the absorbance of NDM-Zn-Ag-TNTs is lower after 533 nm. Consequently, the addition of Ag has improved the visible-light photocatalysis of TNTs. Although the covering layers of Zn^2+^ and NDM decreased the enhancement of the photocatalysis of Ag-TNTs, their photocatalysis ability is still higher than that of bare TNTs over a certain range, and may improve even further after the decomposition of NDM and the delivery of Zn^2+^.

### Release behavior of Zn^2+^

**Figure 7:**
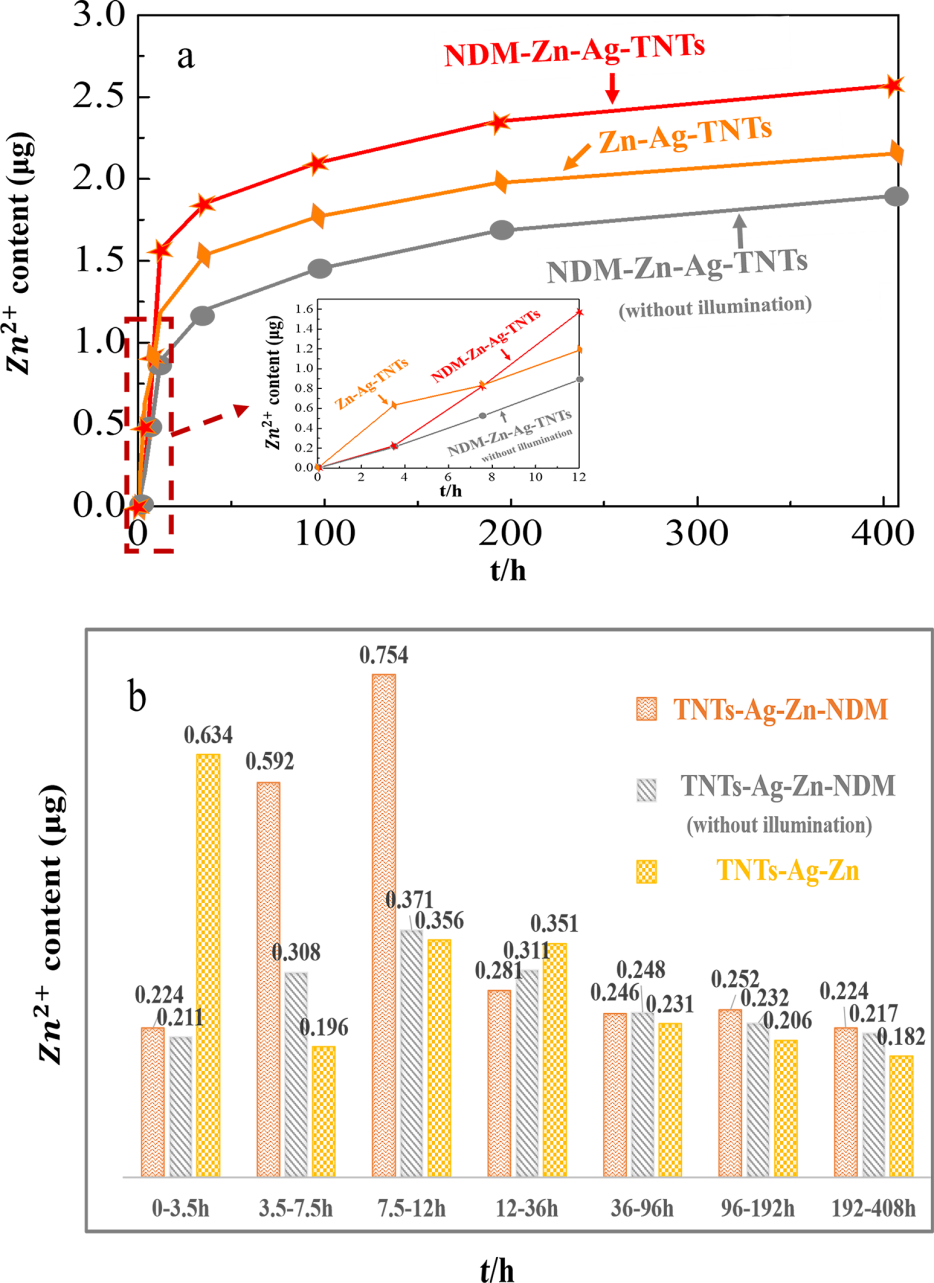
(a) The accumulated drug (Zn^2+^) release with time measured with ICP-AES where the insert is a magnified graph of the drug release process during the initial 12 h: NDM-Zn-Ag-TNTs under illumination (red star-dot line), Zn-Ag-TNTs under illumination (orange diamond-dot line), NDM-Zn-Ag-TNTs without illumination (gray circle-dot line). (b) The amount of drug (Zn^2+^) release of the different samples at given time intervals measured with ICP-AES.

The Zn^2+^ release characteristics under different conditions and various coating layers are presented in Figure 7 where the amount of Zr is measured with inductively coupled plasma atomic emission spectroscopy (ICP-AES). We observe an initial burst release in the initial 12 h followed by a slow release stage from 12 to 408 h thereafter the drug release is evenly sustained after 408 as displayed in Figure 7a. Throughout the 408 hours, the total Zn^2+^ amount released by the sample is 2.573 μg for NDM-Zn-Ag-TNTs under illumination, 2.155 μg for Zn-Ag-TNTs under illumination, and 1.898 μg for NDM-Zn-Ag-TNTs without illumination, respectively. During the burst release stage (shown in the inset of Figure 7a), NDM-Zn-Ag-TNTs, Zn-Ag-TNTs under illumination, and NDM-Zn-Ag-TNTs without illumination showed cumulative Zn^2+^ release amounts of about 1.570 μg, 1.186 μg and 0.890 μg, respectively, accounting for 61.02%, 55.03% and 46.89% of their whole release amount after 408 h. Consequently, the sample with NDM under dark conditions has the lowest release amount at the burst stage and for the whole experiment, indicating that the hydrophobic layer (NDM) plays a role in inhibiting Zn^2+^ release. The sample exhibited the highest accumulated Zn^2+^ release amount after 12 h, demonstrating that visible light could facilitate the release process. The release amount of the different samples at certain time intervals is shown in Figure 7b. Within 3.5 h, the sample without NDM displayed a quicker and less controllable release of 0.634 μg (29.4%) Zn^2+^ as compared to TNTs with NDM under irradiation, which released 0.634 μg (8.7%) Zn^2+^. The results also demonstrate the inhibition behavior of the NDM layer. In the time ranges of 3.5–7.5 h and 7.5–12 h, the sample with NDM releases Zn^2+^ abruptly under irradiation (0.592 μg and 0.754 μg) as shown in Figure 7b, compared with Zn-Ag-TNTs under illumination (0.196 μg and 0.356 μg) and NDM-Zn-Ag-TNTs without illumination (0.308 μg and 0.371 μg), which means that controlled light irradiation could be used to control the drug release. The results suggest that the NDM layer can be rapidly decomposed by the light and then the hydrophilic PBS solution can easily infiltrate the nanotubes to increase the amount of Zn^2+^ released. The hydrophobic layer (NDM) can be applied for the inhibition of drug release, and thereby to extend the course of drug release. Meanwhile, visible light can be used to control drug release via degradation of the polymer coating on the nanotubes and can subsequently accelerate the process of drug release.

Song et al. [14] detected the influence of illumination intensity on the release properties of the amphiphilic and the normal TiO_2_ nanotube layers using the model drug horseradish peroxide (HRP). They also detected the influence of light intensity (dark or under visible light) on the controlled drug delivery, with the release efficiency of amipicillin from the amphiphilic or normal nanotubes detected within a time scale of 120 min [25]. Our previous study [15] using a protein as a model drug and testing within 200 min of release (using the same two-layer nanotubes with different diameters of the first layer and the second layer as prepared in this work) shows that the smaller diameter (60–80 nm) of the top layer can limit the drug delivery and prolong the delivery process. This work focuses on the influence of both the light intensity and the hydrophobic layer of NDM on the release properties of the two-layer nanotubes with the different diameter. Also, the time period is up to 408 hours. The results suggest that the two-layer nanotubes containing Ag nanoparticles with a hydrophobic layer of NDM show the controlled and prolonged release property.

## Conclusion

AgNPs have been successfully decorated on the surface of TNTs (Ag-TNTs) to improve the photocatalysis properties of the TNTs under visible-light irradiation. Then, a NDM hydrophobic layer and ZnO were effectively loaded on the Ag-TNTs. Compared with samples without the hydrophobic layer, the nanotubes coated with NDM, which acts as a drug screen, can further extend the release rate of Zr-based drugs due to the pear-like shape of the nanotube structure. The results also show that visible light can be applied to control the release of drugs by regulating the decomposition of NDM. Evidently, after the decomposition of the hydrophobic layer, the PBS solution can more easily fill the nanotubes, and thus more Zn^2+^ can be released more quickly. Accordingly, this drug release system based on TiO_2_ nanotubes may exhibit a promising application as a localized drug delivery platform.

## Experimental

### Materials

Commercially pure titanium sheets (10 × 10 × 1 mm^3^) were purchased from Luoke Titanium Industry (Chengdu, China). Acetone was purchased from Changlian Chemical (Analytic Reagent, AR, Chengdu, China). Ethanol, dodecanethiol (NDM), ammonium fluoride (NH_4_F), ethylene glycol, toluene, oxydol, NaBH_4_, AgNO_3_, and Zn(AC)_2_ were purchased from KeLong Chemical (AR, Chengdu, China). Other chemical reagents used in these experiments were of analytical grade and were used directly without further purification. Deionized (DI) water was used to clean and prepare aqueous solutions.

### Fabrication of pear-like titania nanotubes

**Figure 8:**
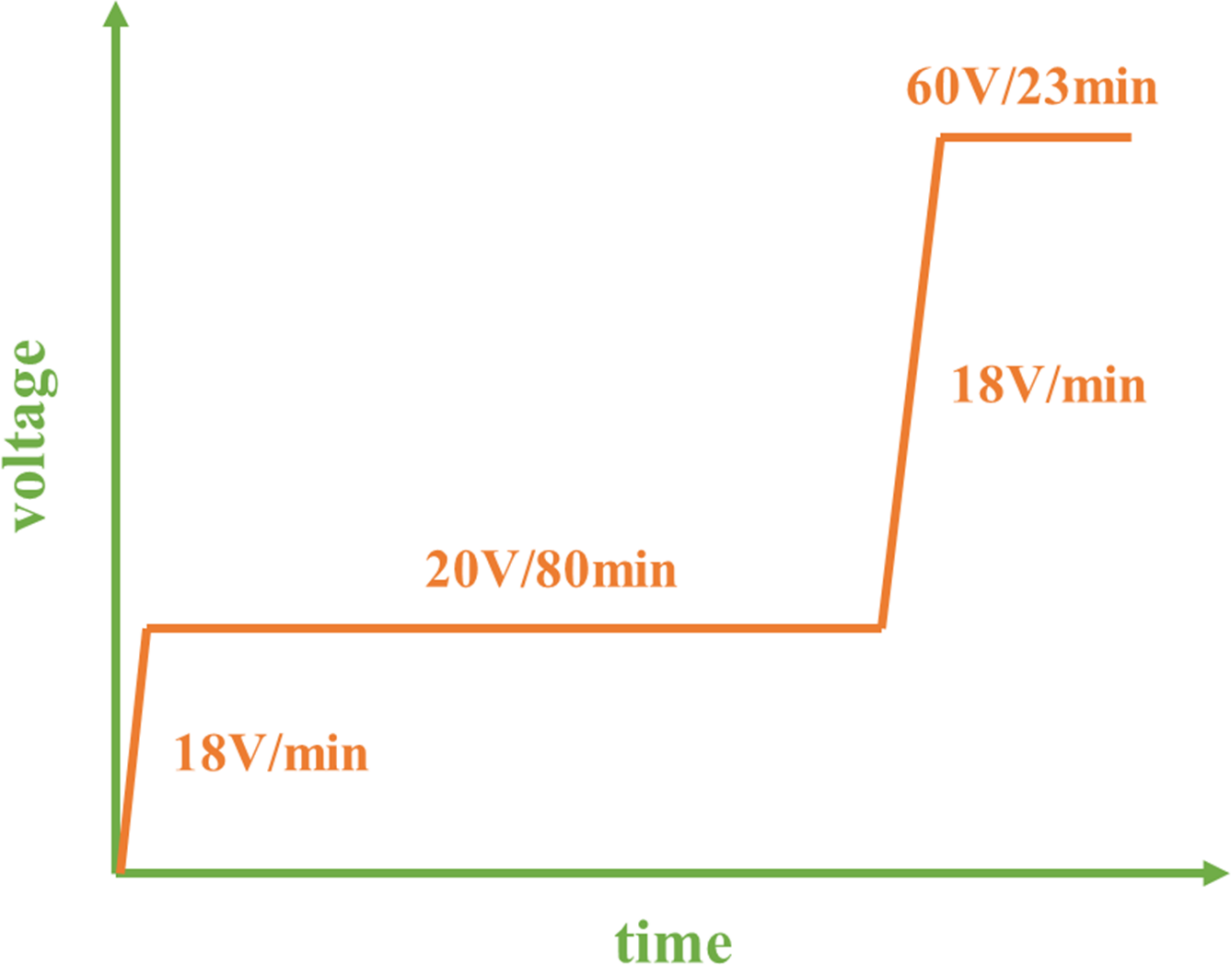
The voltage anodization scheme of the in situ voltage up-anodization process to produce pear-like titania nanotubes.

After degreasing in ethanol and DI water by sonication, the Ti sheets were polished by using metallographic abrasive papers (No. 400, 600 and 800) and further ultrasonically cleaned in the sequence of pure acetone, ethanol and distilled water. Anodic oxidation was conducted using a programmable power supply (Maynuo DC source Meter, Shanghai, China) and a three-electrode configuration with a bicathode composed of two circular titanium plates as counter electrodes. The electrolyte was ethylene glycol containing 6 vol % distilled water and 0.3 wt % NH_4_F and the temperature was maintained at 25 °C. Two-layer nanotubes were fabricated through the in situ voltage up-anodization process [15] in two stages (Figure 8). In the first stage, the voltage was increased from 0 to 20 V at a rate of 18 V/min and anodized at 20 V for 80 min to produce the upper layer. Subsequently, in the second stage, the lower layer was prepared by increasing the voltage from 20 to 60 V at the same rate, and then holding for 23 min. Finally, all samples were cleaned using distilled water in an ultrasonic bath and then desiccated. All the obtained TNT arrays were annealed at 450 °C for 2 h in air atmosphere to form anatase phase.

### Decoration with Ag nanoparticles

Chemical reduction was applied to decorate the nanotubes with Ag nanoparticles (AgNPs). The annealed TNTs were soaked in 4 mL 200 mM AgNO_3_ for 50 min in darkness and then dipped in 6 mL 5 mM NaBH_4_ for another 50 min. The resulting Ag-doped TNTs (Ag-TNTs) were further washed by DI water and dehydrated at room temperature.

### Zn loading and dodecanethiol coating of Ag-decorated titania nanotubes

The procedure of Zn loading into Ag-TNTs was as follows: half of the Ag-TNTs were dipped in 100 mL 0.1 M Zn(AC)_2_ solution for 12 h under yellow fluorescent light-emitting diode (LED) lamp irradiation (λ > 450 nm). Subsequently, the treated TNTs arrays were soaked in 70 mL 0.1 M Zn(AC)_2_ solution and then the samples were treated by hydrothermal reaction in 0.1 M Zn(Ac)_2_ solution for 3 h at 200 °C to fabricate the Zn-loaded Ag-TNTs, which are referred to as Zn-Ag-TNTs throughout this article. Then, a hydrophobic layer was coated onto the tube walls by immersing some of the Zn-Ag-TNTs samples in a 5 mM NDM/toluene solution at room temperature for 24 h in darkness.

### Zn^2+^ release behavior

The Zn-Ag-TNTs were immersed in 5 mL of PBS solution placed in 10 mL centrifuge tubes to wash off the Zn^2+^ substance loosely attached to the surfaces. The sample was placed into plastic centrifuge tubes. Then the surfaces were immersed in 5 mL of PBS at room temperature with orbital shaking. 5 mL of solution was taken after specific interval times (3.5 h, 7.5 h, 12 h, 36 h, 96 h, 192 h and 408 h) and 5 mL of fresh PBS was added to compensate for the solution. The samples were collected periodically for up to 17 days. During the whole process, half of tubes with NDM and half without NDM were irradiated by a yellow fluorescent light-emitting diode (LED) lamp without ultraviolet light (<450 nm), while the other half of tubes with NDM were kept in the dark.

### Materials characterization

The surface morphology and microstructure of the fabricated samples were investigated by scanning electron microscopy (SEM, Hitachi S4800, Japan) and X-ray diffraction (XRD, SHIMADZU 61000, Japan), respectively. The chemical composition of the prepared samples was characterized by energy dispersive X-ray spectroscopy (EDS, Oxford) attached to the SEM. The wettability of the samples was measured by a contact angle meter (JC2000C1; Shanghai, China), and all of the contact angle measurements were conducted in triplicate. The light absorbance of bare TNTs, Ag-TNTs, Zn-Ag-TNTs and NDM-Zn-Ag-TNTs samples ranged from 450 nm to 800 nm and was detected by a UV–vis spectrophotometer (U-3010, Hitachi, Japan). X-ray photoelectron spectrometry (XPS, Escalab 250Xi, USA) of Zn-Ag-TNTs and Ag-TNTs was employed to analyze surface elemental composition and chemical states of the coating film. The data was analyzed for Zn content using inductively coupled plasma atomic emission spectroscopy (ICP-AES, Shanghai-Ins Precise Instrument IRIS Adv.).
